# Patients’ perspective on self-management: type 2 diabetes in daily life

**DOI:** 10.1186/s12913-019-4384-7

**Published:** 2019-08-28

**Authors:** Astrid N. van Smoorenburg, Dorijn F. L. Hertroijs, Tessa Dekkers, Arianne M. J. Elissen, Marijke Melles

**Affiliations:** 1 Faculty of Industrial Design Engineering, Delft University of Technology, Delft, the Netherlands; 2 Department of Health Services Research, Care and Public Health Research Institute, Faculty of Health, Medicine and Life Sciences, Maastricht University, Maastricht, the Netherlands; 3 Department of Public and Occupational Health, Amsterdam Public Health research institute, Amsterdam UMC, Vrije Universiteit Amsterdam, Amsterdam, the Netherlands

**Keywords:** Patient preferences, Lifestyle, User-centred design, Chronic care, Context mapping

## Abstract

**Background:**

The number of type 2 diabetes mellitus (T2DM) patients and related treatment costs are rapidly increasing. Consequentially, more cost-effective and efficient strategies for the treatment of T2DM are needed. One such strategy is improving patients’ self-management. As patients are more and more expected to self-manage their disease, it is important to provide them with suitable self-management support. This way, success of self-management will increase and complications and related costs of T2DM can be reduced. Currently, self-management support is developed mainly from the perspective of health professionals and caregivers, rather than patients. This research focused on gaining a better understanding of patients’ perspectives on self-management and support.

**Methods:**

Semi-structured interviews, preceded by preparatory assignments, were conducted with ten patients with T2DM treated in Dutch primary care.

**Results:**

We found that patients experience ‘active’ self-management when recently diagnosed. As time progresses and no problems occur, patients do not experience their disease-related behaviour as self-management. Diabetes has ‘just’ become part of their daily life, now including new routines taking diabetes into account.

**Conclusions:**

With this knowledge, support solutions can be designed and implemented that better fit the needs, preferences and abilities of patients with T2DM.

**Electronic supplementary material:**

The online version of this article (10.1186/s12913-019-4384-7) contains supplementary material, which is available to authorized users.

## Background

Diabetes mellitus is a growing healthcare challenge. Currently, 415 million adults worldwide have diabetes, a number that is expected to rise to 642 million by the year 2040 [1]. Of all patients, approximately 90% has type 2 diabetes mellitus (T2DM). Patients with T2DM have a high risk of developing diabetes-related complications, such as cardiovascular diseases, retinopathy and kidney disease. The global spending on direct health care costs of T2DM and its related complications was estimated to be International Dollar (ID) 795 to 1404 billion in 2015, and is expected to increase to ID 997 to 1788 billion in 2040 [1]. Therefore, it is of vital importance to develop and implement more cost-effective and efficient strategies for the treatment of T2DM.

In the Netherlands, diabetes care is of high quality as indicated by the excellent Euro Diabetes Index-scores concerning, amongst other, multidisciplinary collaboration and coordination between healthcare providers [2]. Moreover, approximately 70% of Dutch patients with T2DM has adequate glycaemic control (glycated haemoglobin (HbA1c) levels ≤60 mmol/mol), indicating that blood sugar levels are within acceptable range [3]. Nevertheless, these patients do not seem to fully benefit from the evidence-based guidelines for treatment of T2DM, which are currently highly standardised and focused on regular face-to-face consultations with health professionals rather than on supporting patients’ self-management at home. Findings from previous research suggest that patients with adequate glycaemic control are able to maintain this level of control when the frequency of consultations with health professionals is reduced, for example from 3-monthly to 6-monthly monitoring [4]. As complications of T2DM are strongly associated with an unhealthy lifestyle [5–7] focusing on self-management, including lifestyle change, may be a more efficient treatment strategy for healthcare providers as well as patients.

Self-management is defined as the active participation of patients in their treatment [8]. According to Corbin and Strauss [9], self-management comprises three distinct sets of activities: (1) medical management, e.g. taking medication and adhering to dietary advice; (2) behavioural management, e.g. adopting new behaviours in the context of a chronic disease; and (3) emotional management, e.g. dealing with the feelings of frustration, fright, and despair associated with chronic disease. Since T2DM is a chronic disease and patients only see health professionals a few times a year, patients themselves need to be in control of all these aspects for the remainder of time.

Self-management support is one of the essential components of the Chronic Care Model, a well-known guide to improve the management of chronic conditions [10]. Optimal support of patients’ self-management targets all three sets of tasks set out by the Corbin and Strauss framework [9] and stimulates providers and patients to use a collaborative approach to ‘identify problems, set priorities, establish goals, create treatment plans and solve issues along the way’ [11]. Previous research has shown that successful support of self-management of patients with T2DM can have a positive impact on their lifestyle and, ultimately, result in improved health outcomes [12–15]. However, international comparative research [16] also shows that self-management support remains relatively underdeveloped in most countries. Moreover, it is often developed from the perspective of health professionals and care providers, rather than patients. Clear insight into patients’ perspectives on self-management and how this is currently supported could contribute to the development of solutions that better fit the needs, preferences and abilities of patients. It is expected that adequate self-management support improves health outcomes and efficiency of care [17–19]. Therefore, the objective of this study is to gain a better understanding on the perspectives of patients with T2DM regarding self-management (support).

### Methods

This study is part of the Dutch research project PROFILe (PROFiling patients’ healthcare needs to support Integrated, person-centred models for Long-term disease management). The aim of the PROFILe project is to determine optimal treatment strategies for subgroups of patients with T2DM with similar care needs, preferences and abilities, taking into account both clinical and non-clinical aspects [20]. As part of the PROFILe project, opportunities for improving self-management support for patients with T2DM were explored in this study. Qualitative research was performed by conducting in-depth interviews preceded by preparatory (*‘sensitising’*) assignments, in order to get detailed insights into individual experiences of patients [21]. No ethical approval was needed for the study; as the participants were not physically involved in the research and the questionnaires were not mentally exhausting, the study was not subject to the Dutch Medical Research (Human Subject) Act. All patients participating in the study gave written informed consent.

### Participants

Previous research from the PROFILe project suggests that there is a relatively large subgroup of patients with recently diagnosed T2DM (5 ≤ years), who are expected to benefit from increased self-management support and decreased dependence on health professionals [22]. Therefore, patients from this specific group were targeted in this research. Accordingly, patients were included if they: 1) were diagnosed with T2DM no longer than five years ago; 2) made use of diabetes-related care provided by Dutch primary care; and 3) had a stable, adequate glycaemic control (i.e. HbA1c ≤60 mmol/mol). Participants were recruited in the period from March to April 2017 by email via the Dutch Association for Diabetes (in Dutch: *Diabetes Vereniging Nederland),* through announcements in diabetes-related Facebook groups, by inviting people present at a ‘Diabetes Café’ (a monthly meeting for people with diabetes) and via personal contacts. Patients received a monetary reimbursement for participating in the research. Participation was voluntary, and all participants provided informed consent.

### Study design

**Fig. 1:**
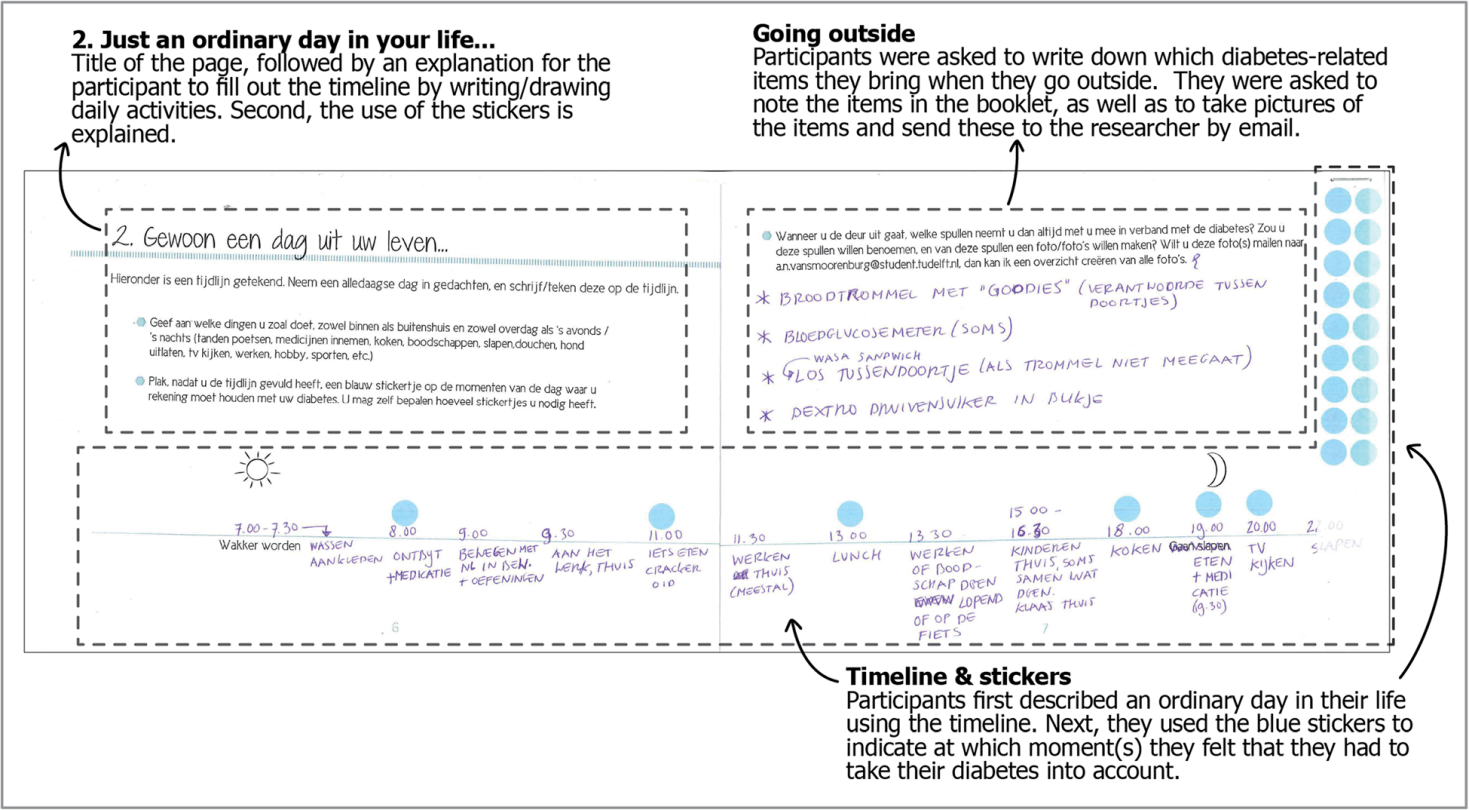
Example page from the sensitising booklet (in Dutch). Patients filled out a timeline and questions about ‘An ordinary day in their life’ (‘*Gewoon een dag uit uw leven’*). The blue stickers were used to indicate moments in the day where the participant felt he or she had to take diabetes into account. During the interview, the participant was asked to explain how diabetes was taken into account in these moments, and how the participant experienced this

Patients were invited to prepare themselves for the interviews by filling out so-called *sensitising booklets* [23]. The aim of the exercises in the booklets was to trigger participants to reflect on their experiences with self-management of diabetes. Topics addressed in the booklet included *‘Just an ordinary day in your life...’, ‘Type 2 diabetes’, ‘Information’,* and *‘Manager of my diabetes’*. An example of one of the pages from the sensitising booklet is shown in Fig. 1. Patients filled out the booklets at home for 5 days in a row prior to the interview, focusing on a different topic and taking about 15 min each day. The use of sensitising booklets is a well-known tool within the domain of user-centred design research, i.e. a design research approach which emphasises user involvement throughout the design (research) process. Using sensitizing booklets enables the researcher to quickly engage with the interviewee, prepares the interviewee for the interview, and allows for elaboration on specific topics that were mapped prior to the interview. This way, a deeper (tacit or latent) layer of information about the perspective of the patient can be addressed during the interviews [23].

Next, semi-structured face-to-face interviews were conducted by the first author from March to April 2017. The researcher prepared a set of interview questions aligned with the exercises in the sensitising booklet. For example “*Which medication do you take because of diabetes?”,* “*Could you explain the role your diabetes played during the time you gave a blue sticker on your time line*?” “*What is the difference regarding diabetes when you are at home and when you are on-the-go?”* and “*How could you become more / less a manager of your diabetes?”.* The interviews ended with the question *‘In your opinion, which aspects constitute ‘Diabetes in your daily life?’*. These aspects were written down and ranked by the participant according to impact on daily life (scale 1 (least) – 5 (most)). The full list of interview questions is presented in Additional file 1. Each interview took about 60 min and was performed in the local language (Dutch) at the participants’ house, or another location of their preference. The interviews were voice recorded for analysis.

### Analysis

The interviews were analysed in four steps. First, voice recordings of the interviews were listened back, while making notes of the answers of all participants for each of the five topics of the booklet. In the second step these notes were condensed to create statements within each of the topics according to a general inductive approach [24]. For example, the notes “I cannot do something spontaneaously anymore, because I always have to take diabetes into account and make adjustments accordingly.”, “Because of diabetes, I need to prevent hastiness and stress.”, and “I cannot do unexpected things, because of the diabetes.” were condensed to the statement “Diabetes requires a regular schedule.”. Third, the statements were discussed with the co-authors and categorized as concerning: 1) elements of self-management (e.g. exercising, knowledge, being in control); 2) characteristics of the disease and treatment (e.g. type of medication, diet, use of blood sugar level meter); and 3) characteristics of the attitude towards the disease (e.g. acceptance, consequences, role of health professional vs. role of patient). Taking into account the objective of this paper, only the results of the first category will be presented. In step 4, we defined the patient’s perspective towards self-management by defining the different themes that contribute to this perspective and clustering the statements in the self-management category.

## Results

### Participant characteristics

Sixteen people applied for participation in the study. Ten people (62.5%) met all inclusion criteria and were included. Table 1 shows an overview of participants’ background characteristics. Participants were mostly female (70%) with a mean age of 53.4 years (SD 11.2) and relative recent diagnosis of T2DM, ranging from four months to circa four years ago. Mean HbA1c was 50.7 (SD 6.5) mmol/mol. All participants were treated for T2DM by a general practitioner (GP) and practice nurse specialized in diabetes care at the GP practice. Seven interviews took place at the participant’s home, two interviews were performed at the participant’s work office, and one interview was done in a restaurant.

**Table 1:** Overview of background characteristics of participants

Diagnosed since	Age range	Gender	HbA1c
< 2 years	35–55	Female	49 mmol/mol
		Female	49 mmol/mol
		Female	56 mmol/mol
	> 55	Male	49 mmol/mol
		Male	59 mmol/mol
> 2 years	35–55	Female	44 mmol/mol
		Female	45 mmol/mol
	> 55	Female	41 mmol/mol
		Female	55 mmol/mol
		Male	60 mmol/mol

### Patients’ perspective on self-management

The perspective of patients with T2DM on self-management is organised in relation to self-management as ‘diabetes in daily life’, ‘active’ self-management, the impact of the disease on daily life, and lastly it is described how patients currently experience support in self-management.

### Self-management as ‘diabetes in daily life’

Self-management is a term which is commonly used by health professionals. However, most participants in our study did not experience their behaviour since the diagnosis of T2DM as ‘self-management’. Rather, they felt they dealt with their daily life as it is now, just as every other person with or without T2DM. In other words, from the perspective of the participants, having diabetes did not suddenly make a person more of a ‘manager’: “Only the moment of hearing the diagnosis was difficult, because it is not nice to hear you need to change *your comfortable daily life which you have been used to for so long. But, apart from that, diabetes is not difficult; you just need to learn how to deal with it*.”

Participants did not often experience problems caused by deteriorated glycaemic control, and therefore did not consider themselves as having to actively self-manage their disease. They ‘just’ made adjustments and compromises regarding their habits and routines. One volunteer of the Dutch Association for Diabetes, who monthly organises a Diabetes café said as well: “*From a patients’ perspective, there is no conscious self-management, it is just dealing with diabetes in daily life*.”

### ‘Active’ self-management

**Fig. 2:**
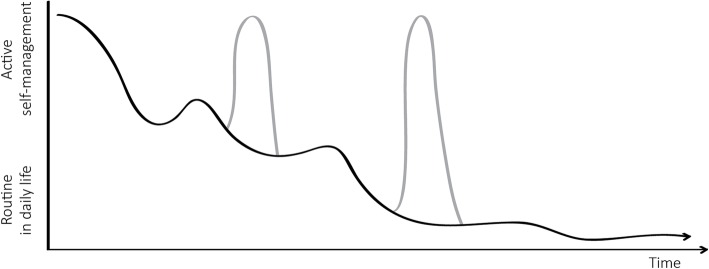
Over time, active self-management changes into routine in daily life. When problems occur, patients shift back to active self-management (grey peaks)

Although self-management was generally described as *diabetes in daily life*, participants also mentioned that if glycaemic control was no longer stable, a need for active self-management emerged. They described that at such times, actions were required to prevent complications. Looking at this ‘active’ self-management over time (Fig. 2), it can be seen that when recently diagnosed, patients felt an active need to manage. However, over time, new lifestyles became part of their routine in daily life and were no longer experienced as active self-management.

### The impact of diabetes on daily life

All patients mentioned that T2DM influenced their daily life. Yet, the impact of T2DM on daily activities was greater for some patients than for others. For example, regarding the effort it takes to minimise the intake of carbohydrates a day, one patient mentioned: *“It is a struggle for me every time I see my husband and children eating a cookie at night”*. Another participant, however, did not feel she was missing out on appetizing food when seeing her family eating food which she could not eat anymore: *“It is just a different way of cooking and eating, I can still have delicious meals and snacks”*.

Whether patients considered diabetes to have a large impact on their daily life also seemed to influence their acceptance of diabetes and the new lifestyle. Some patients felt that diabetes had to be taken into account at all times. One patient mentioned *“There is no choice. The health professional gives advice, but you have to do the work and decide what to eat and drink and what not.”* This patient felt confronted with T2DM every time and had not yet accepted it as much as others who successfully made adjustments to old habits or developed new ones that take diabetes into account.

Since patients experienced *diabetes in daily life* rather than *self-management*, aspects which influence diabetes in daily life were investigated. The aspects (scored by the participants on a five-point scale) that had the most impact (4 or 5 out of 5) on the daily life of T2DM patients were categorised and are shown in Table 2. Three categories were identified: lifestyle changes, medication, and knowledge/control. According to the participants, all aspects are related: for example when ‘Exercising’, the timing, amount and intensity must be tuned with ‘Food and drinks’ in order to keep blood sugar levels within range. As the timing and of medication also influences these aspects, patients’ expressed the need for a regular schedule. To account for these different aspects patients felt required to be in control, and to have sufficient knowledge to keep control.

**Table 2:** Aspects named by the participants having most impact (4 or 5 out of 5) on daily life of T2DM patients

Categories	Aspects named by participants to have most (4 or 5 out of 5) impact on daily life	Explanations	Quotes from participants
Lifestyle changes	Food and drinks	Eating and drinking is necessary throughout the day, but the amount of carbohydrates always needs to be taken into account.	*“I used to always count carbohydrates, but not anymore. Now I just know what I can and cannot eat a day.”*
	Exercising	It is healthy to exercise, but timing, amount and intensity of exercising must be attuned to the intake of carbohydrates (or the other way around).	*“Walking with the dog is fun to do! It motivates and supports me in exercising.”*
	Regular schedule	The daily life of patients with T2DM requires a regular schedule, because otherwise blood sugar levels will be out of control.	*“The need for always taking into account my schedule for eating, that is annoying”*
Medication	Medication	Different types of medication can be prescribed for T2DM, depending on HbA1C level of the patient. Every type of medication requires tuning with food intake and exercising, and the need for medication can be reduced by having a healthy lifestyle.	*“I do not need any medication; I only pay very good attention to my lifestyle to keep the HbA1c level within good range.”*
Control & knowledge	Being in control	Patients want to be in control of their blood sugar levels, and therefore in control of the diabetes throughout the day. Nevertheless, this is not always possible, for example, emotions influence blood sugar levels as well, which can be very difficult to control.	*“By doing measurements with this blood glucose level meter, I start to learn to control diabetes, because I can actually see the effect of my behaviour.”*
	Knowledge	Patients feel like they need a lot of knowledge about T2DM and how to best deal with it, so they know what to adjust and do in their daily life.	*“You are overwhelmed by all new information, but still you feel like you don’t know anything about it.”*

### Experienced support for self-management

Participants mentioned very specific things that made them feel supported. For example, with regard to exercising, patients felt supported by their dog or children. However, patients were not able to mention specific causes for not feeling supported. For example, concerning exercise, they mentioned a lack of support in motivation. Overall, patients felt supported in self-management in some ways, but mainly felt as if they had to find out everything about living with diabetes on their own. In their view, health professionals provide medical advice, but could not explain how to deal with T2DM in daily life.

## Discussion

The daily care for type 2 diabetes mellitus (T2DM) mostly comes down to the person suffering from it. To maintain adequate glycaemic control, patients with T2DM have to make many decisions and fulfil complex care activities every day [25]. Respondents in our study mentioned a need to gain knowledge, be in control, adapt their diet, exercise, maintain a regular schedule, and adhere to complex medication regimes. However, in fulfilling these responsibilities, they did not view themselves as actively participating in their treatment, at least not continuously. Rather, what is conceptualized in the literature as self-management was viewed by respondents as ‘simply’ dealing with diabetes in their daily life, which became an integrated part of their (new) daily routines. Thus, it seems that patients with a recent diagnosis (< 5 years ago) and stable, adequate glycaemic control have limited needs for professional support as long as initial support is provided during the first few weeks after diagnosis. This is in line with previous research indicating that patients who perceive their illness as stable have different needs for support than patients who experience their disease as episodic or progressively deteriorating [26]. An unpredictable course of illness can cause feelings of lower self-efficacy, i.e. patients might experience their self-management as unsuccessful and, as a result, feel a greater need for support [27, 28]. Although overall, respondents did not experience themselves as actively managing their diabetes, they did identify two time points of active self-management during their illness course, particularly in the period after diagnosis and when problems occurred. With regard to support for their self-management, patients expressed that they did not feel optimally supported, which is in line with findings from previous studies [16, 29]. However, they had difficulties in describing what is lacking, suggesting that they do not know what exactly is missing or how support could be improved.

Self-management needs to be supported in order to more successfully treat T2DM [30]. This research explored the concept of self-management from the patients’ perspective. This person-centred perspective is valuable, as patients are expected to be in control of management of T2DM in daily life. Therefore, outcomes of this research can be used to develop tools and strategies that support self-management in a way that better fits the needs of T2DM patients. The development of tools and strategies from the perspective of the user (i.e. patient) is known as ‘user-centred design’. Solutions developed in a user-centred way may improve acceptance of interventions as they closely match patients’ needs and expectations of support. It may also improve cost-effectiveness of the intervention, as costly implementation of features that patients do not want or cannot use is avoided [31]. Our findings suggest two aspects that are important to consider in developing user-centred self-management support interventions for patients with T2DM. First, it is important to provide support at the right moments, i.e. when patients experience a need for support due to changes in their daily routines or changes in their health. Two such moments were identified in our study: the period directly after diagnosis and at instances when problems occur (glycaemic control deteriorates). As to the latter, previous studies have also shown that as patients’ self-rated health deteriorates, their self-management support needs increase [26]. In addition to physical limitations, such as pain and fatigue, which further complicate self-management, deterioration of health can cause feelings of loss of control, and disappointment that previous self-management strategies have failed. At such moments, patients might be more open to professional support to make sustainable behavioural change to maintain glycaemic control, and prevent – or at least postpone – the debilitating long-term complications of insufficient glycaemic control. Second, it is important to provide support for relevant element(s), i.e. which the person with T2DM experiences as challenging in daily life: food and drinks, exercising, regular schedule, medication, being in control, and/or knowledge. By taking into account these specific topics when developing tools and strategies, patients will be better supported and therefore better able to successfully self-manage their disease.

An important strength of this research is its focus outside medical context. The research addressed the participant as a person (with T2DM), not as a patient. Also, interviews were done by a researcher, not a medical specialist, and took place at the participants’ house or another location of their preference, instead of a healthcare facility. This way, participants expressed they felt comfortable in sharing their experiences regarding T2DM and self-management. Participants mentioned that within the medical context, they fear being criticised on the way they cope with the disease as health professionals mostly focus on HbA1c values and less on the T2DM-related issues of the patient. Another strong aspect of this research is the use of sensitising booklets for elicitation of the participants’ perspectives. Patients were triggered to think about their personal experiences regarding management of and dealing with T2DM prior to the interview. Therefore, the researcher could touch upon a deeper layer of information during the interviews.

This study explored self-management and self-management support needs from the perspective of patients with T2DM rather than health professionals. We focused particularly on the subgroup of patients with a recent diagnosis and stable, adequate glycaemic control, for whom self-management support may be a more cost-effective- and efficient treatment approach than provider-led care. However, patients who have not yet achieved stable, adequate glycaemic control may have different support needs, which should be explored in further detail. Furthermore, the sample size was sufficient for the current qualitative study, as the aim was to get detailed insights into the experiences of individuals. Nevertheless, to assess the generalizability of findings, it is important to replicate the current study with a larger sample of patients. This may require different methodology as well. The general inductive approach used in the current study provides only a first description of important themes related to patients’ perspectives on self-management and support. However, this methodology is less applicable to theory and model building [24]. To develop an overall representative theory of self-management from the patient perspective other qualitative methods such as grounded theory may be more appropriate. Moreover, 7 out of 10 participants were female. This does not represent the 50% male / 50% female ratio of patients with T2DM in the Netherlands. Finally, the outcomes of this research do not yet provide insight in what patients currently miss regarding support in self-management. In order to further improve self-management support, additional research is needed on this aspect.

Two moments have been indicated by this study which are most optimal for providing support; when recently diagnosed and when problems occur. Future research can further explore the differences and similarities for providing support to people in these different moments. It is possible that different strategies for support would be best for each moment.

## Conclusions

This research focused on the needs of a specific patient group; T2DM with stable, adequate glycaemic control. This population has not been researched before, and therefore new insights are generated for this target group specifically. Outcomes of this study can now be further explored in a broader view, but these first insights already indicate the need for a more individualised approach to support patients with T2DM and a stable, adequate glycaemic control. The current guidelines for treatment of T2DM are too standardised and lack personalised support in specific aspects as dietary behaviour, exercising, scheduled rhythm, medication, being in control, and knowledge. Improving support for self-management will have a positive effect on patients’ lifestyle and health outcomes, motivate them to maintain successful self-management, and ultimately limit complications and related costs.

## Additional file

Additional file 1: Interview questions. (PDF 569 kb)

## Data Availability

The interview records and sensitising booklets generated and analysed during the current study are not publicly available to protect participant confidentiality, but are available from the corresponding author on reasonable request.

## Abbreviations

GP: General practitioner
HbA1c: Glycated haemoglobin
T2DM: Type 2 diabetes mellitus
