# Supplementary material for: Patients’ perspective on self-management: type 2 diabetes in daily life
Source: BMC Health Serv Res. 2019 Aug 28;19:605. doi: 10.1186/s12913-019-4384-7 (PMC6714441; doi:10.1186/s12913-019-4384-7)
Supplement: Supplementary file 1 — Interview questions. (PDF 569 kb) [file 12913_2019_4384_MOESM1_ESM.pdf]

Appendix A

Individual interview (following sensitising booklet)

Date:

Participant:

Informed consent 2x:

Recording device:

Take a brief look at the sensitising booklet.

1. *Was everything clear when filling out the booklet?*

2. *Booklet: Who are you?*
   2.1. Which medication do you use because of diabetes? (pills/ insulin shots/ …)
   - How many times a day/week?
   - What is this medication for? (Blood sugar level, cholesterol, …)

3. *2. Just an ordinary day in your life…*
   3.1. Could you please explain to me for the blue stickers what that situation is like, and what is the role of diabetes in that moment?
   - Could you please demonstrate this for me? (make pictures)
   - How does this involve/affect the people living with you?
   - Which topics are there? Are there any more?
   - What would this moment look like without diabetes?
   - Does sometimes also something happen during the night because of diabetes?
   - Which moments do you dislike most? Why?
   - Do you think this is similar to other people with type 2 diabetes? What is similar and what could be different?

   3.2. Could you please explain to me when and how you use these materials? (make pictures if not already sent by participant)
   - Write down the names of the materials
   - What would happen if you did not have these materials with you?
   - Did you ever forget one of these materials? How did that go? How did you manage to solve this?

   3.3. Regarding diabetes, what is the difference between being at home, or not being at home?
   - Could you explain to me what sometimes goes wrong when not being at home? And while being at home? (Examples of last time this happened?)
   - Is it more difficult to take into account diabetes when being at home, or not at home? Why?

   3.4. Could you explain to me which moments in the past two weeks were most difficult for you to deal with diabetes (at home or not)?
   - What did happen then?

- Who were there when it happened?
- How did it end? How was it solved?
- Could it have been prevented?

4. Booklet: 3. Type 2 diabetes
    4.1. P8.: Could you please explain to me why you wrote down these?
        - In what way does this influence your lifestyle / social activities / emotions?
        - Which are positive to you? (mark)
        - Which are negative to you? (mark)
        - Do you intend to stimulate the positive ones? How?
        - Do you intend to prevent the negative ones? How?
    4.2. P8.: Could you please mark the most important (max. three) aspects for me using this pen?
        - Why is this/ are these most important to you?
    4.3. P9.: How would you experience diabetes differently after this would be changed? (for every point)
        - What would be different then?
        - What would be the effect of the change?
        - What do you mean by more simple/ easier / ….?

5. *Booklet: 4. Information*
    5.1. The last time you were looking for information, how did this go?
        - Which resources did you consult? (why only one, or why multiple?)
        - Why did you choose these resources?
        - Why in this order?
        - What type of information were you looking for? (numbers, experiences of peers, asking for advices, ….)
        - How do you think other people look for information?
    5.2. When do you consult friends and relatives, and when do you consult other resources? Why?
        - Could you please give an example of that?
        - What would happen if you could not consult these people anymore?
        - What would happen if you could not consult these other resources anymore?
    5.3. Is there anything you would like to change about this way of collecting information? Why/Why not?
        - For answers to your questions?
        - For information of which you do not even know it is there?
        - From whom/what should this information come? How often?

6. *Booklet: 5. 'Manager' of my diabetes*
    6.1. After you were diagnosed with type 2 diabetes, what was the process of setting up your treatment plan like?

- Who were involved here?
- To what extend were you involved in this process?
- Would you have liked to be more or less involved in this process? Why?
- In general, what is described in the treatment plan? (goals, medication, …?)
- Is this treatment plan still used, for example when visiting the GP?
- What do you think of this treatment plan?

6.2. In what way could you become more/less of a manager of your diabetes?

- What should change for that? How? Why?

6.3. P13.: for what is written down; Could you please describe/explain these situations to me?

- Is this similar to the current situation, or not (yet)?
- Medication: only taking medication, or as well deciding about the dose?
- Help: Who/What should provide this help? (Friend/relative, health professional, product, …) Why?
- Health professional: Which health professional should be in charge of this? Why?
- Health professional: How would your health professional feel about this do you think? Why?
- What would you do, if there would not be any support provided to you?
- What would happen if a health professional would not be in charge of this?

7. *Lastly, I would like to summarise all this. Therefore, I made this card.*
   What is, according to you, 'Diabetes in daily life'?

7.1. Together with the participant, write down the 'modules'.

7.2. Write down the influence (on daily life), scale 1-5 (1 = least, 5 = most)

7.3. For which of these elements do you currently feel supported? How?

- Preparation / registration / feedback / alarming / …

7.4. By whom/what do you feel supported?

7.5. When/In what situation do you feel supported?

7.6. For which of these elements do you currently <u>not</u> feel supported? How?

- Preparation / registration / feedback / alarming / …

7.7. By whom/what do you feel <u>not</u> supported?

7.8. When/In what situation do you <u>not</u> feel supported?

7.9. In which aspects would you prefer to be supported? (why, how, by whom/what, when,…?)

7.10. For which aspects you do not need any support? Why?

8. *Make a 'profile picture' of the participant & hand over the VVVbon. Is it okay if I contact you again later on during this project for a following research?*
